# A hybrid multi-objective optimization of functional ink composition for aerosol jet 3D printing via mixture design and response surface methodology

**DOI:** 10.1038/s41598-023-29841-0

**Published:** 2023-02-13

**Authors:** Haining Zhang, Zhixin Liu, Shuai Yin, Haifeng Xu

**Affiliations:** 1 School of Information Engineering, Suzhou University, Suzhou, 234000 China; 2 School of Mechanical and Aerospace, Nanyang Technological University, Singapore, 639798 Singapore; 3 China Aerospace Times Feihong Technology Co., Ltd., Beijing, 100854 China

**Keywords:** Characterization and analytical techniques, Electronic devices, Electronic properties and materials

## Abstract

The limited electrical performance of microelectronic devices caused by low inter-particle connectivity and inferior printing quality is still the greatest hurdle to overcome for Aerosol jet printing (AJP) technology. Despite the incorporation of carbon nanotubes (CNTs) and specified solvents into functional inks can improve inter-particle connectivity and ink printability respectively, it is still challenging to consider multiple conflicting properties in mixture design simultaneously. This research proposes a novel hybrid multi-objective optimization method to determine the optimal functional ink composition to achieve low electrical resistivity and high printed line quality. In the proposed approach, silver ink, CNTs ink and ethanol are blended according to mixture design, and two response surface models (ReSMs) are developed based on the Analysis of Variance. Then a desirability function method is employed to identify a 2D optimal operating material window to balance the conflicting responses. Following that, the conflicting objectives are optimized in a more robust manner in the 3D mixture design space through the integration of a non-dominated sorting genetic algorithm III (NSGA-III) with the developed ReSMs and the corresponding statistical uncertainty. Experiments are conducted to validate the effectiveness of the proposed approach, which extends the methodology of designing materials with multi-component and multi-property in AJP technology.

## Introduction

Aerosol jet printing (AJP) is emerging as a transformative three-dimensional (3D) printing technology to fabricate high-resolution and flexible micro-electronic devices^1,2^. Compared with conventional fabrication techniques, such as etching, photolithographic and electroplating^3^, AJP can significantly reduce chemical waste and simplify manufacturing process^4^, while lowering fabrication costs^5^. Therefore, it has been widely adopted in an electronic manufacturing industry to fabricate advanced microelectronic components^6–8^. However, due to the relatively high printed line resistivity of AJP, the electrical performance of the microelectronic devices produced by AJP is limited, such as the low sensitivity/gauge factor of the printed resistive sensors for temperature/strain measurement, which will restrict the wide application of AJP technology. This is because of the gaps and defects in the deposited nanoparticle structure, such as granular boundaries and structural defects^9^. Consequently, the connectivity between metallic nanoparticles is seriously damaged, and the printed metallic lines show relatively high electrical resistivity compared with bulk metal materials, undermining the electrical performance of the printed electronics significantly. Despite optimizing the sintering conditions (temperature and technique) can improve the surface morphology of printed lines, achieving a resistivity of less than 2 times of the bulk silver resistivity^10,11^, it is also important to reduce the resistivity of printed lines by enhancing the connectivity in the nanoparticle structure before the post-printing process. Besides, as the low ink printability of a functional ink tends to induce nonequilibrium aerodynamic interaction within the printhead, the conductive lines will be printed with inferior morphological features^12^, which will further degrade the electrical performance of the produced electronics^13^.

Due to the extreme aspect ratio, carbon nanotubes (CNTs) demonstrate advantages as bridges to connect the defects/granular boundaries in the printed conductive lines, which will enhance the conductivity of printed electronics by improving the electrical contacts between the formed particles and thermal expansion coefficients between printed patterns and substrates^14^. Therefore, there have been various studies on incorporating CNTs into nanoparticle functional inks to provide superior inter-particle connectivity and improve the electrical properties of aerosol jet printed electronic components^15^. Besides enhancing electrical properties, the conductive-on-demand capability of printed films can be achieved based on tailorable and controllable CNTs loading^16^. However, as the conductivity of CNT is relatively lower than that of metallic nanoparticle functional inks, the performance of CNTs as conductivity-enhancing fillers depends on the relative concentration compared to the percolation level^17^. Therefore, further studies aim at optimizing the relative composition between CNTs and nanoparticle functional ink for conductivity enhancement are required. On the other hand, as a deposited line is the fundamental element of aerosol jet printed electronic components^18^, various machine learning methods and empirical approaches were adopted to optimize the printed line quality^19–21^, which will be beneficial to the obtained electrical performance^13^. Despite these studies help to promote the application of AJP technique from a certain aspect, there is a need to further improve the overall electrical performance under the dual optimization of printed line conductivity and printing quality simultaneously.

Despite increasing the diversity of ink composition helps AJP technology fabricate versatile micro-electronic devices, there are few systematic studies on optimizing materials with multi-component and multi-property in AJP technology. For example, a novel CNTs/silver nanoparticle ink was formulated to enhance the conductivity of aerosol jet printed lines^9^, but the ink composition was optimized by empirical approach, and the printed line quality needs further evaluation. On the other hand, a platinum/ethylene glycol/polyvinylpyrrolidone functional ink was synthesized to achieve the low resistivity for aerosol jet printed lines^22^. As the optimal printed line quality was obtained by optimizing the process parameters under specific ink composition, the influence of ink components on resistivity needs further investigation. Under such circumstances, a novel hybrid multi-objective optimization method is proposed to formulate the correlations between the functional ink composition (silver ink, CNTs and ethanol) and printed line electrical/morphological properties (printed line resistivity/quality), and optimize the conflicting relationship between low electrical resistivity and high printed line quality simultaneously. Experiments are conducted to validate the effectiveness of the proposed method for conductivity and morphology optimization of aerosol jet printed lines. Compared with traditional methods, the proposed method is developed based on the theory of design of experiments, statistical modeling and multi-objective optimization, which facilitates the optimization of ink compositions in a data-driven manner. Therefore, the proposed method is not limited to specific ink materials, but can be applied to the optimization of different ink components suitable for AJP.

## Experiments and methods

### Ink selection

It is important to select nanoparticle functional ink with high boiling point and high viscosity co-solvent for aerosol jet printing, which helps obtain printed lines with good edge definition by reducing the coffee ring effect. Moreover, due to the equipped ultrasonic atomizer, the range of viscosity for the adopted functional ink is limited^23^. On the other hand, it is essential to adopt single-walled CNTs with a low number of wall defects, otherwise the defect can cause deformation of the CNTs along the tube axis, which could be problematic during aerosol transportation. And, the length of CNTs should be much shorter than the droplet to avoid clogging the nozzle. In this research, Clariant® (producer headquarter: Switzerland) nanoparticle silver ink was used as the original functional ink because of its suitable solvents (water and Ethylene glycol) and ink properties (density: 1.35 kg/m^3^, surface tension: 0.036 N/m, viscosity: 8.3 cP). Besides, Nanolab® (producer headquarter: USA) NINK 1100 single-walled CNTs ink was used for mixture design because of its suitable CNT length distribution (average length: 1300 nm, standard deviation: 615 nm) and long-term stability (solvent: water)^24^. Additionally, the purchased Tedia® (producer headquarter: USA) ethanol was used as received without any further purification due to its high purity (99.7%).

### Experimental process and setup

Focus ratio (FR), plate temperature, deposition speed and ink stabilization time are crucial process parameters of AJP technology. Specifically, a reasonable FR is helpful to balance aerodynamic interaction within the nozzle tip, resulting in narrower lines with more distinct edges. To ensure the overall printability of AJP under different ink compositions, the shielding gas flow rate (SFR), atomizer flow rate (AFR) and hence the FR (SFR/AFR) were experimentally determined in this research. On the other hand, despite a hot plate is helpful to remove the excess solvent during printing, as this study investigated the influence of volume fraction of each composite on the deposited line morphology, the plate temperature was kept at room temperature to ensure the stability of the deposited line morphology. Moreover, as high roughness and high overspray lines may be produced due to significant interaction between high gas flow rates and high deposition speed, a relatively low deposition speed (1 mm/s) was adopted to ensure printing quality. Additionally, as the instable shielding gas flow (SF) and atomizer flow (AF) can cause nonequilibrium aerodynamic interaction within the nozzle tip, resulting in irregular line morphology, a 3-min stabilization time was taken between each set point change to obtain a stable flow of material. Therefore, the experimental setup of AJP process is defined as shown in Table 1, and the gas flow rates were in standard cubic centimeters per minute (sccm).

**Table 1:** Experimental setup of the AJP process.

	Experimental conditions						Process parameters		
Atomization current	Tip diameter	Ink temperature	Plate temperature	Stabilization time	Sintering temperature	Sintering time	SFR	AFR	Print speed
0.45 mA	150 μm	20 °C	Room temperature	3 min	200 °C	2 h	60 sccm	42 sccm	1 mm/s

**Figure 1:**
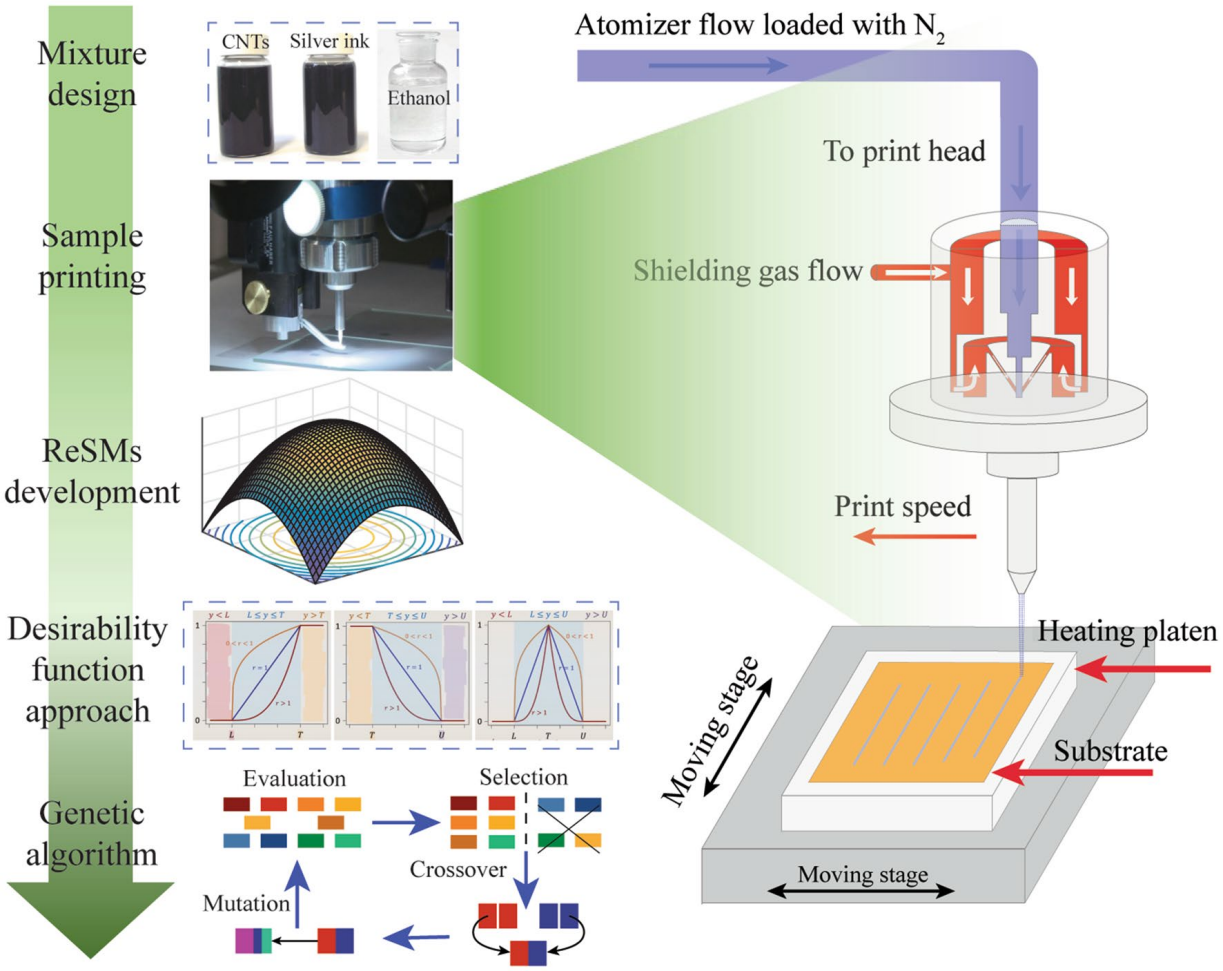
Main experimental process of the proposed optimization approach.

Figure 1 illustrates the main experimental phases of the proposed optimization approach. Before printing, surfaces of polyimide substrates were subjected to bath cleaning for five minutes and corona plasma ultrasonic treatment for three minutes successively, which helps improve the wetting behavior of the polyimide substrate during the printing process. Then, according to mixture design, the test materials were formed by blending nanoparticle silver ink, CNTs ink and ethanol. To ensure a good deposition and stability of the printing process, the mixed ink was mechanical stirring for 10 min and ultrasonic treatment for 20 min at 25 ºC successively prior to printing to ensure uniform dispersion of particles in the ink medium. The same mixing procedure was applied for all inks to maintain the comparability of the obtained experimental results. Following that, a single pass of line was printed onto a polyimide substrate based on the developed ink, and five line samples were fabricated successively by repeating each experimental point five times. After sample fabrication, the deposited morphological characteristics and the corresponding printed line resistivity were extracted for developing the response surface models (ReSMs). Subsequently, a desirability function approach was employed to determine a 2D optimal operating material window to balance the conflicting responses. Finally, the conflicting objectives were optimized in a more robust manner in the 3D mixture design space through the integration of genetic algorithm (GA) with the developed ReSMs and the corresponding statistical uncertainty.

In this research, as the operating conditions were maintained, and each experimental point was repeated 5 times, the repeatability of the deposited line morphology can be evaluated. As shown in Fig. S1, despite there is some randomness due to the aerodynamic interaction within the printhead, the main morphological characteristics of the deposited lines are retained in the repeated experiments, indicating the acceptable stability of the AJP process. Additionally, to verify the negligible influence of coffee-ring effect on the printed line morphology, the cross-sectional profiles of printed lines were measured 4 h after deposition at room temperature. As shown in Fig. S2, the line profile presented negligible concave shape at the central area, confirming that the coffee-ring effect had a negligible effect on printed line morphology^25^.

### Morphological characterization of printed line samples

The morphological characterization of printed line samples is described in Supplementary Figs. S3, S4. Figure S3a illustrates that the morphological characteristics of deposited line samples, including printed line overspray and edge roughness can be evaluated based the determined mean lines. Specifically, based on image processing, the original image (see Fig. S3b) is discretized into a grey image (see Fig. S3c). Then, after image denoising (see Fig. S3d), the width of mean lines can be measured by the detected actual line edge

1 $$\overline{w}=\frac{1}{N}\sum_{i=1}^{N}{w}_{i}$$

where $N$ represents the number of columns of a discretized line edge, ${w}_{i}$ denotes the corresponding line width of the discretized *i*-th column. Thereafter, the overall printed line edge roughness ${E}_{R}$ can be evaluated by

2 $${E}_{R}=\sqrt{\frac{1}{2N}\sum_{i=1}^{N}\left({ER}_{upper,i}^{2}+{ER}_{lower,i}^{2}\right)}$$

where ${ER}_{i}$ denotes the printed line width variation of the *i*-th column correspondingly. And, the printed line overspray ${O}_{SP}$ in the original image (see Fig. S3c) can be measured as follows

3 $${O}_{SP=}\frac{1}{2N}\sum_{i=1}^{N}\left({OS}_{upper,i}+{OS}_{lower,i}\right)$$

where ${OS}_{i}$ denotes the corresponding length between the actual line edge and the overspray micro-droplets of the discretized *i*-th column. In this study, the overall printed line quality is evaluated by normalizing the weight sum of overspray ${O}_{SP}$ and edge roughness ${E}_{R}$ of deposited line samples in a mixture design space.

4 $${O}_{M}={{w}_{SP}\times {O}_{SP}+w}_{ER}\times {E}_{R}$$

where ${O}_{M}$ denotes the weight sum of printed line the morphological features, ${w}_{ER}$ and ${w}_{SP}$ denote the weights of deposited line edge roughness and overspray, respectively. Supplementary Table S1 illustrates the representative microscopic images of printed lines and the corresponding quantified printed line quality.

Due to the linear relationship between the length and resistance of aerosol jet printed lines, Ohm’s law is employed as follows to describe the length dependence of resistance for the printed line samples^13^

5 $${R}_{T}=\rho \frac{L}{S}$$

where the printed line resistance ${R}_{T}$ is measured by the four-point probe method, $S$, $L$ and $\rho$ are the cross-sectional area, length and resistivity of a deposited line sample, respectively. In this research, a linear array of five line samples ($L=1.5$ cm) was printed onto the substrate, the cross-sectional area $S$ of each line sample as shown in Fig. S4 was measured 5 times and averaged for further resistivity calculation.

### Mixture design in a response surface modeling (RSM)

Considering the variability of the functional ink properties, it is important to design the experiments suitable for obtaining feasible compounds of different components and evaluating their influence on printed line electrical/morphological properties. In this case, a mixture design is adopted in this research to investigate the individual influence and interactions of different ink components, and a RSM is employed to investigate the cause-effect relationship between the input variables and the outputs based on the constructed mixture design experimental points^26^. Due to the component volume fraction dependence assumption of the response, the construction of experimental points should be restricted to the upper limit (${u}_{i}$) and lower limit (${l}_{i}$) of each component volume fraction (${x}_{i}$).

6 $$0\le {l}_{i}\le {x}_{i}\le {u}_{i}\le 1, \sum_{i=1}^{n}{x}_{i}=1$$

where $n$ is the number of components in mixture design research. The experimental points are determined by the following rules^27^:

1. Determine the maximum range $R$ of the component volume fractions by
  7 $$R=1-\sum_{i=1}^{n}{l}_{i}$$
2. Determine the upper limit of the quasi-component volume fraction by
  8 $${u}_{i}^{{^{\prime}}{^{\prime}}}=min\left\{\left({u}_{i}^{{^{\prime}}{^{\prime}}}-{l}_{i}\right)/R,1\right\}$$
3. If ${u}_{i}^{{^{\prime}}{^{\prime}}}=1$, set $\left(0, \dots , 1,\dots ,0\right)$ as the vertices
4. If ${u}_{i}^{{^{\prime}}{^{\prime}}}<1$ and ${u}_{i}^{{^{\prime}}{^{\prime}}}+{u}_{j}^{{^{\prime}}{^{\prime}}}>1 \left(i\ne j\right)$, set $\left(0,\dots ,{u}_{i}^{{^{\prime}}{^{\prime}}},0,\dots {u}_{i}^{{^{\prime}}{^{\prime}}},0\dots ,0\right)$ as the vertices
5. If $${u}_{i}^{{^{\prime}}{^{\prime}}}+{u}_{j}^{{^{\prime}}{^{\prime}}}\le 1$$ and $${u}_{i}^{{^{\prime}}{^{\prime}}}+{u}_{j}^{{^{\prime}}{^{\prime}}}+{u}_{k}^{{^{\prime}}{^{\prime}}}>1 \left(i\ne j\ne k\right)$$,
  set $\left(0,\dots ,{u}_{i}^{{^{\prime}}{^{\prime}}},0,\dots {u}_{i}^{{^{\prime}}{^{\prime}}},0\dots ,0, 1-{u}_{i}^{{^{\prime}}{^{\prime}}}-{u}_{i}^{{^{\prime}}{^{\prime}}},0\dots 0\right)$ as the vertices as well
6. The center points and check blends of the polyhedron can be further calculated based on the determined vertices^28^.

**Figure 2:**
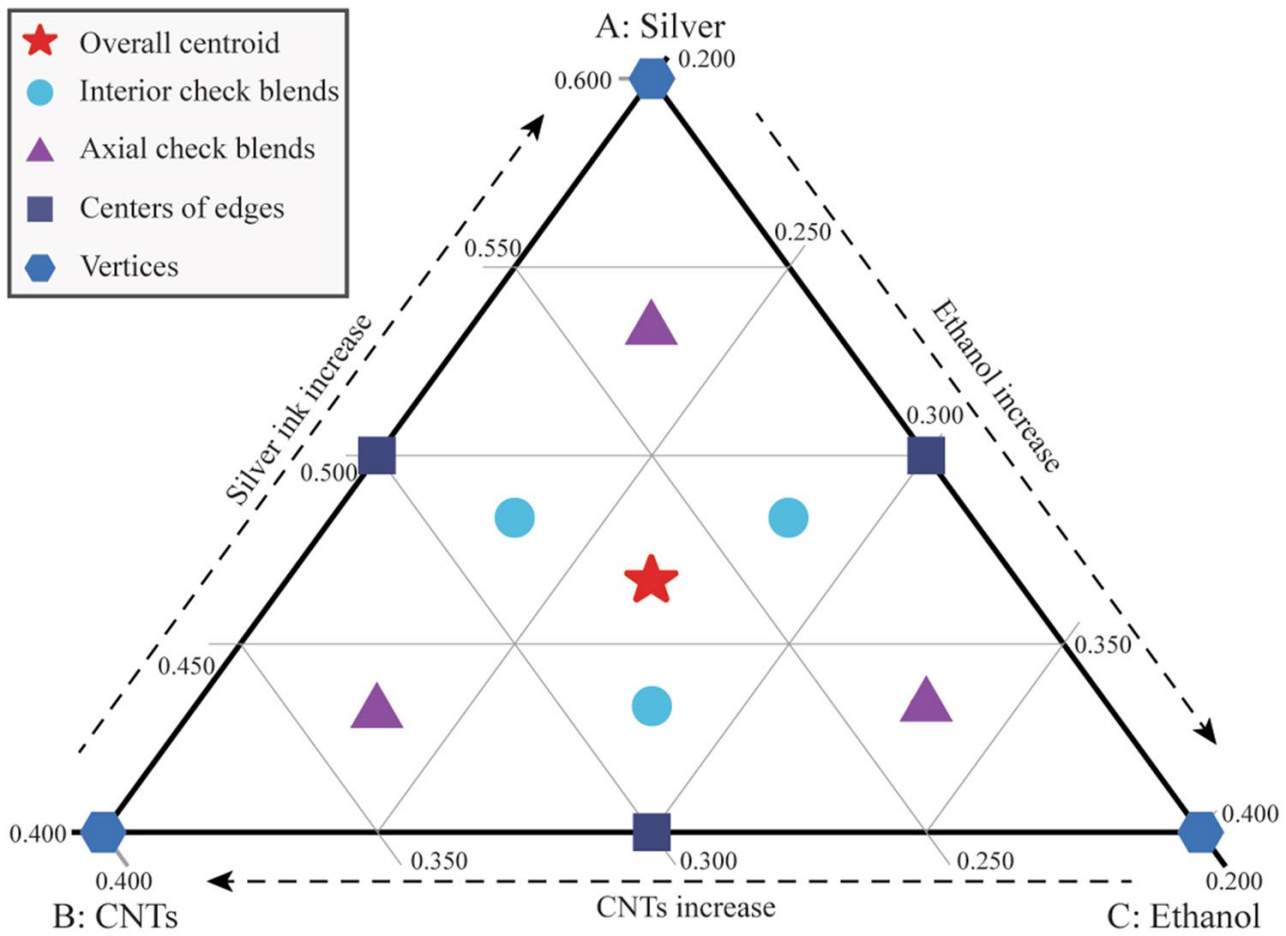
Designed experimental points in a mixture design space of AJP.

In this study, the ranges of the volume fraction of silver ink, CNTs ink and ethanol are 40–60%, 20–40% and 20–40%, respectively. Figure 2 shows the experimental points designed by a mixture design algorithm in this research; the overall centroid will be replicated three times to evaluate the system reproducibility^29^. The obtained actual experimental points are shown in Supplementary Table S2.

After obtaining the experimental results, the quadratic ReSMs will be employed to formulate the correlations between the functional ink composition and printed line electrical/morphological properties in the mixture design space.

9 $$Y=\sum_{i=1}^{n}{\beta }_{i}{x}_{i}+\sum_{1\le i<j}^{n}{\beta }_{ij}{x}_{i}{x}_{j}$$

where ${\beta }_{i}$ and ${\beta }_{ij}$ are constants determined by ANOVA and the least square method.

### Statistical uncertainty

According to mixture design and the corresponding RSM, a quadratic model can be derived to formulate the relationship between input variables ${\varvec{x}}={({x}_{1}, {x}_{2}, \dots , {x}_{m})}^{T}$ and the output $y({\varvec{x}})$ as follows

10 $$y\left({\varvec{x}}\right)={g({\varvec{x}})}^{T}{\varvec{\theta}}$$

where *m* refers to the number of the inputs, ${\varvec{g}}\left({\varvec{x}}\right)$ and ${\varvec{\theta}}$ are defined as

11 $${\varvec{g}}\left({\varvec{x}}\right)={\left(1,{x}_{1},\dots ,{x}_{m},{x}_{1}^{2},\dots ,{x}_{m}^{2},{x}_{1}{x}_{2},\dots {x}_{m-1}{x}_{m}\right)}^{T}$$

12 $${\varvec{\theta}}={\left({\theta }_{0},{\theta }_{1},\dots ,{\theta }_{m},{\theta }_{11},\dots ,{\theta }_{mm},{\theta }_{12},\dots ,{\theta }_{m-1,m}\right)}^{T}$$

Based on the developed ReSMs, a (1−α)% confidence interval (CI) is proposed to quantify the prediction uncertainty statistically^30^

13 $$\left(1-\alpha \right)\% \mathbf{C}\mathbf{I}=\left[y({\varvec{x}})\pm {t}_{\frac{\alpha }{2},n-p}\widehat{s}\sqrt{{{\varvec{g}}({\varvec{x}})}^{T}{\left({{\varvec{X}}}^{T}{\varvec{X}}\right)}^{-1}{\varvec{g}}({\varvec{x}})}\right]$$

where $\left(1-\alpha \right)\% \mathbf{C}\mathbf{I}$ refers to a range that the average output $y({\varvec{x}})$ should be within (1−α)% of the time, $\mathbf{X}$ represents an expanded model matrix, ${t}_{\alpha /2,n-p}$ refers to the *t* distribution of (1−α/2) quantile with (*n−p*) degrees of freedom, *p* refers to the number of predictors, *n* refers to the number of experiments, and $\widehat{s}$ refers to the estimated standard deviation.

In this research, the statistical uncertainty σ of the developed ReSMs is evaluated by the 95% CI as follows

14 $$\sigma ={t}_{\frac{\alpha }{2},n-p}\widehat{s}\sqrt{{{\varvec{g}}({\varvec{x}})}^{T}{\left({{\varvec{X}}}^{T}{\varvec{X}}\right)}^{-1}{\varvec{g}}({\varvec{x}})}$$

### A desirability function approach

A desirability function approach can convert the multiple outputs (printed line resistivity/quality) into a single output function^31^, thus optimizing the conflicting objective functions simultaneously. Specifically, each response variable function ${y}_{i}$ is first transformed into an individual desirability ${d}_{i}$ varying in the range of $\left[0, 1\right]$, where ${d}_{i}=0$ demonstrates the range of response ${y}_{i}$ is unacceptable, while ${d}_{i}=1$ means the optimal objective of the individual output ${y}_{i}$ is achieved. To minimize an individual response, the desirability ${d}_{i}$ is quantified based on a Smaller-The-Better (STB) criteria;

15 $${d}_{i}=\left\{\begin{array}{cc}0& {y}_{i}>U\\ {\left(\frac{{y}_{i}-U}{L-U}\right)}^{r}& L\le {y}_{i}\le U\\ 1& {y}_{i}<L\end{array}\right.$$

Conversely, a Larger-The-Better (LTB) criteria will be utilized for desirability quantification if the response is maximization type;

16 $${d}_{i}=\left\{\begin{array}{cc}0& {y}_{i}<L\\ {\left(\frac{{y}_{i}-L}{U-L}\right)}^{r}& L\le {y}_{i}\le U\\ 1& {y}_{i}>U\end{array}\right.$$

where $r$ is specified by users for importance adjustment, $U$ and $L$ denote the upper limit and lower limit of an individual output function, respectively^32^.

Then, the overall desirability $D$ of the $n$ conflicting objectives can be evaluated by combining each individual desirability ${d}_{i}$ as follows

17 $$D={\left({\left({d}_{1}\right)}^{{w}_{1}}{\left({d}_{2}\right)}^{{w}_{2}}\dots {\left({d}_{n}\right)}^{{w}_{n}}\right)}^{1/\sum {w}_{i}}$$

where $n$ denotes the number of individual outputs in the mixture design, ${w}_{i}$ refers to a user-specified weight corresponding to the $i$-th desirability function.

### A non-dominated sorting genetic algorithm

Despite a desirability function approach can efficiently optimize the conflicting objectives by scalarizing the multiple output responses into a single response function, the identified single solution will be significantly affected by the user-specified desirability weights. Therefore, identifying a group of Pareto-optimal points as alternative solutions will help to optimize the conflicting targets more objectively.

A non-dominated sorting genetic algorithm III (NSGA-III) is an important type of GAs for multi-objective optimization. Different from many classic selection methods adopted in GAs, such as tournament selection and roulette wheel selection, NSGA-III can optimize the conflicting objectives by identifying a set of Pareto-optimal points rather than a single solution, which will be more objective and efficient^33^. In addition, unlike previous NSGAs^34^, as NSGA-III proposes a reference point based method for non-dominated sorting and selection, the population diversity of the determined Pareto-optimal front solutions can be further improved. Therefore, NSGA-III is employed in this study to simultaneously evaluate a set of generations in the search space rather than a single point.

Because of the contradiction between printed line resistivity and printing quality, a multi-objective optimization algorithm is required to optimize the conflicting objectives. To promote the robustness and efficiency of the optimization process, a statistical/probabilistic model will be superior to other approaches as both predictive response and prediction uncertainty can be jointly considered in the adopted multi-objective optimization method^35^. In this case, the ReSMs and the corresponding statistical uncertainty are jointly driven with a NSGA-III to systematically optimize conflicting printing results. Supplementary Fig. S5 shows the proposed flow chart for optimizing functional ink composition in 3D mixture design space. We consider the volume fraction of silver ink, CNTs ink and ethanol as the input variables, and set printed line resistivity (${y}^{(LR)}$) and printed line quality (${y}^{(LQ)}$) as the outputs. In this research, the two conflicting output responses are optimized in terms of minimization

18 $${\mathrm{Obj}}_{1}={y}^{(LR)}+{\lambda }_{1}{\sigma }^{(LR)}$$

19 $${\mathrm{Obj}}_{2}=\frac{1}{{y}^{\left(LQ\right)}}+{\lambda }_{2}{\sigma }^{(LQ)}$$

where $\uplambda =\left[1.6, 2.9\right]$ represents the optimization coefficient to balance the model prediction $y$ and the corresponding model uncertainty $\sigma$. Supplementary Fig. S6 shows the chromosome encoding pattern adopted in NSGA-III, and Supplementary Table S3 summarizes the overall system settings of the proposed framework.

## Results and discussion

### Measurement of printed line electrical/morphological properties

**Figure 3:**
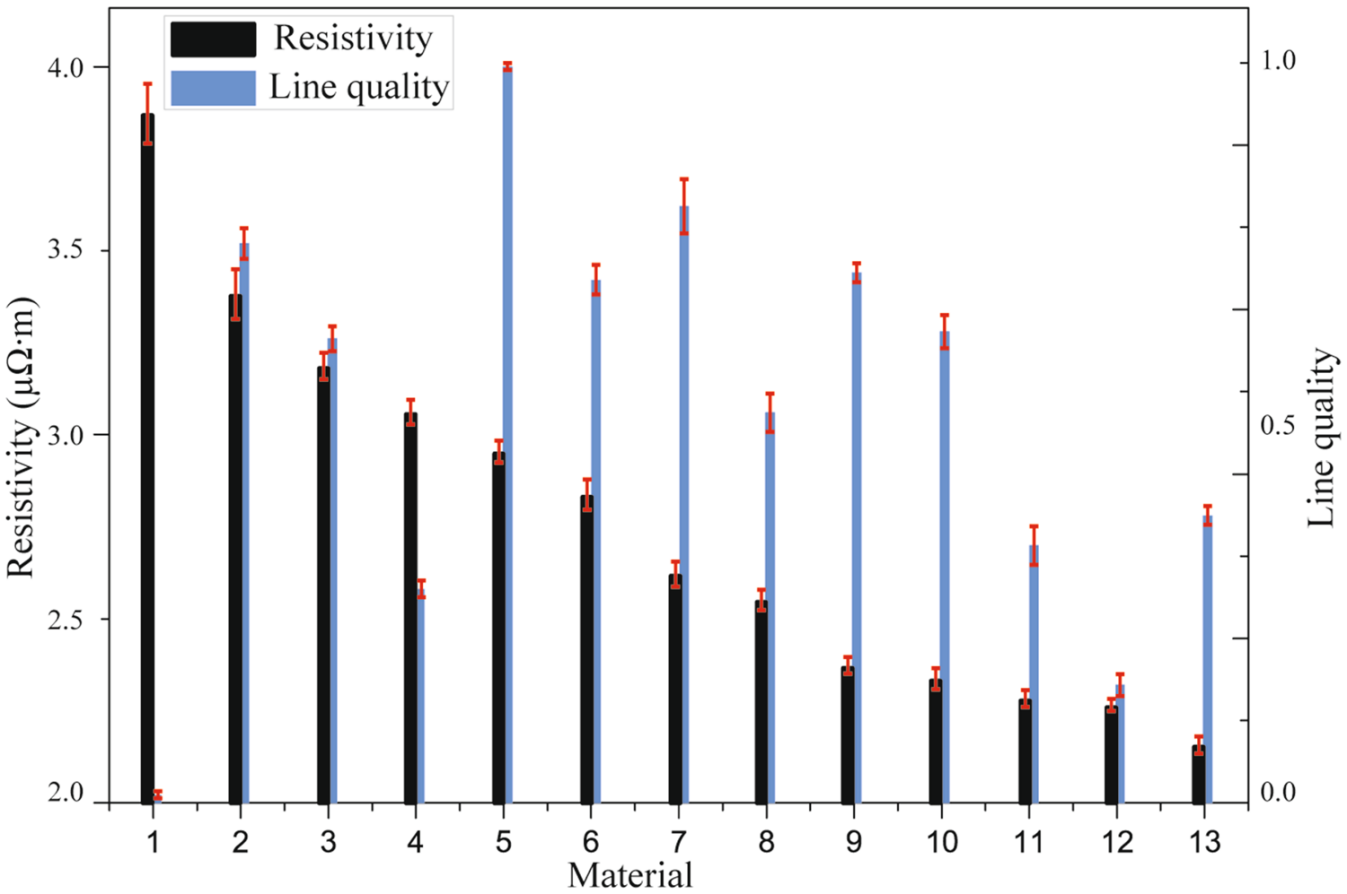
Printed line resistivity/quality of various ink compositions.

The measured printed line resistivity/quality are listed in Table 2, where the material M0 is original functional ink without the incorporation of CNTs and ethanol. Generally, mixing nanoparticle silver ink with CNTs contributes to lower printed line resistivity^36^ which is demonstrated in the obtained experimental results (see materials M9, M10, M11, M12 and M13 in Fig. 3). However, the overall decreasing trend cannot be maintained even with the increased volume fraction of CNTs (see materials M1, M4 and M6 in Fig. 3). Likewise, materials M6, M10, M11 and M13 show that mixing nanoparticle silver ink with increased ethanol helps improve the printed line quality, which may be mainly attributed to the enhanced printability by the incorporation of ethanol^37^. Also, compared to materials M1, M4 and M12, materials M5, M7 and M9 confirm that higher volume fraction of ethanol contributes to better printed line quality. However, further increasing the volume fraction of ethanol cannot keep the overall improvement tendency of the printed line quality (see material M2 in Fig. 3). This is because the volume fraction of each composite cannot be changed independently while the total volume of the mixed material remains unchanged. Consequently, the interaction between the individual ingredient may have a significant impact on mixture design, and the improvement of printed line conductivity/quality will be suppressed as exhibited in the above observations. Therefore, it is important to analyze individual influence and associate interaction of different ink compositions on deposited line electrical/morphological properties.

**Table 2:** Experimental results based on mixture design.

No	Material	Resistivity (µΩ⋅cm)	Line quality
/	M0	3.35	0.63
1	M1	4.09	0
2	M2	3.54	0.76
3	M3	3.32	0.63
4	M4	3.18	0.29
5	M5	3.06	1
6	M6	2.93	0.71
7	M7	2.69	0.81
8	M8	2.61	0.53
9	M9	2.41	0.72
10	M10	2.37	0.64
11	M11	2.31	0.35
12	M12	2.29	0.16
13	M13	2.17	0.39

### ANOVA of the mixture design derived models

In this research, the quadratic ReSMs were utilized to formulate the cause-effect relationship between the influencing parameters (volume fractions of ink components) and output responses (printed line resistivity/quality) statistically. R1, R2 refer to the printed line resistivity and the overall printed line quality. As A, B, C denote the volume fraction of silver ink, CNTs and ethanol, respectively, the interaction between different ink compositions is represented by AB, AC and BC. Specifically, the ANOVA results of the high F-values and low P-values as shown in Table 3 demonstrate the significance of the developed ReSMs. Besides, the high Adeq. precision values and high R-squared values as described in Table 3 reveal the statistical significance of the developed ReSMs as well^38^.

**Table 3:** The ANOVA results of the mixture design derived models.

Factors	R1		R2	
	F value	P value	F value	P value
Model	36.82	< 0.0001	19.21	0.0006
Linear Mixture	69.07	< 0.0001	35.55	0.0002
AB	27.65	0.0012	6.32	0.0402
AC	7.01	0.0331	9.22	0.0190
BC	8.53	0.0223	7.69	0.0276
R^2^	0.9634		0.9321	
Adj. R^2^	0.9372		0.8835	
Adeq. precision	19.196		13.361	

**Figure 4:**
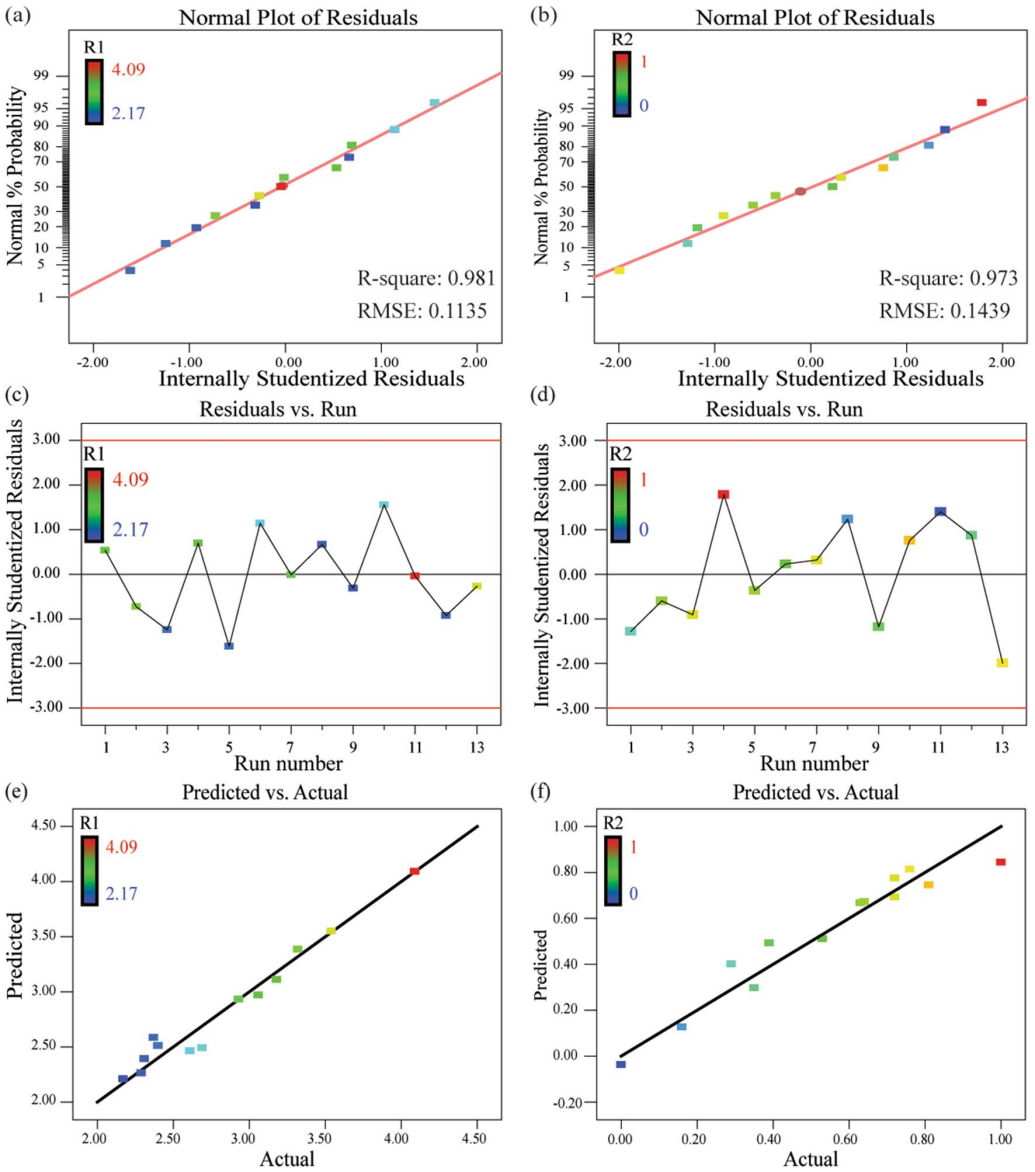
The ANOVA results of the derived ReSMs. (**a**, **b**) Normal plots of residuals for printed line resistivity and printed line quality, respectively. (**c**, **d**) Effects of the run orders on ReSMs residuals of printed line resistivity and printed line quality, respectively. (**e**, **f**) Predicted responses versus actual responses of mixture design for printed line resistivity and printed line quality, respectively.

As the quantified residuals for the model outputs are linearly distributed along the reference line with less deviation, the modeling accuracy is considered to be statistically acceptable (see Fig. 4a, b)^39^. And, the effects of the run orders on the developed ReSMs can be excluded based on the quantified correlations between model residuals and run orders (see Fig. 4c, d). Moreover, Fig. 4e, f) demonstrate that the cause-effect relationship between the volume fraction of each composite and the printed line resistivity/quality is fully captured by the derived ReSMs. Hence, the developed cause-effect relationship with coded units for mixture design is described as follows

20 $$\mathrm{R}1=2.26\mathrm{A}+4.09\mathrm{B}+3.55\mathrm{C}-3.14\mathrm{AB}-1.58\mathrm{AC}-1.74\mathrm{BC}$$

21 $$\mathrm{R}2=0.13\mathrm{A}-0.038\mathrm{B}+0.81\mathrm{C}+1.01\mathrm{AB}+1.22\mathrm{AC}+1.11\mathrm{BC}$$

### Identification of a 2D optimal operating window of functional ink composition via a desirability function approach

**Figure 5:**
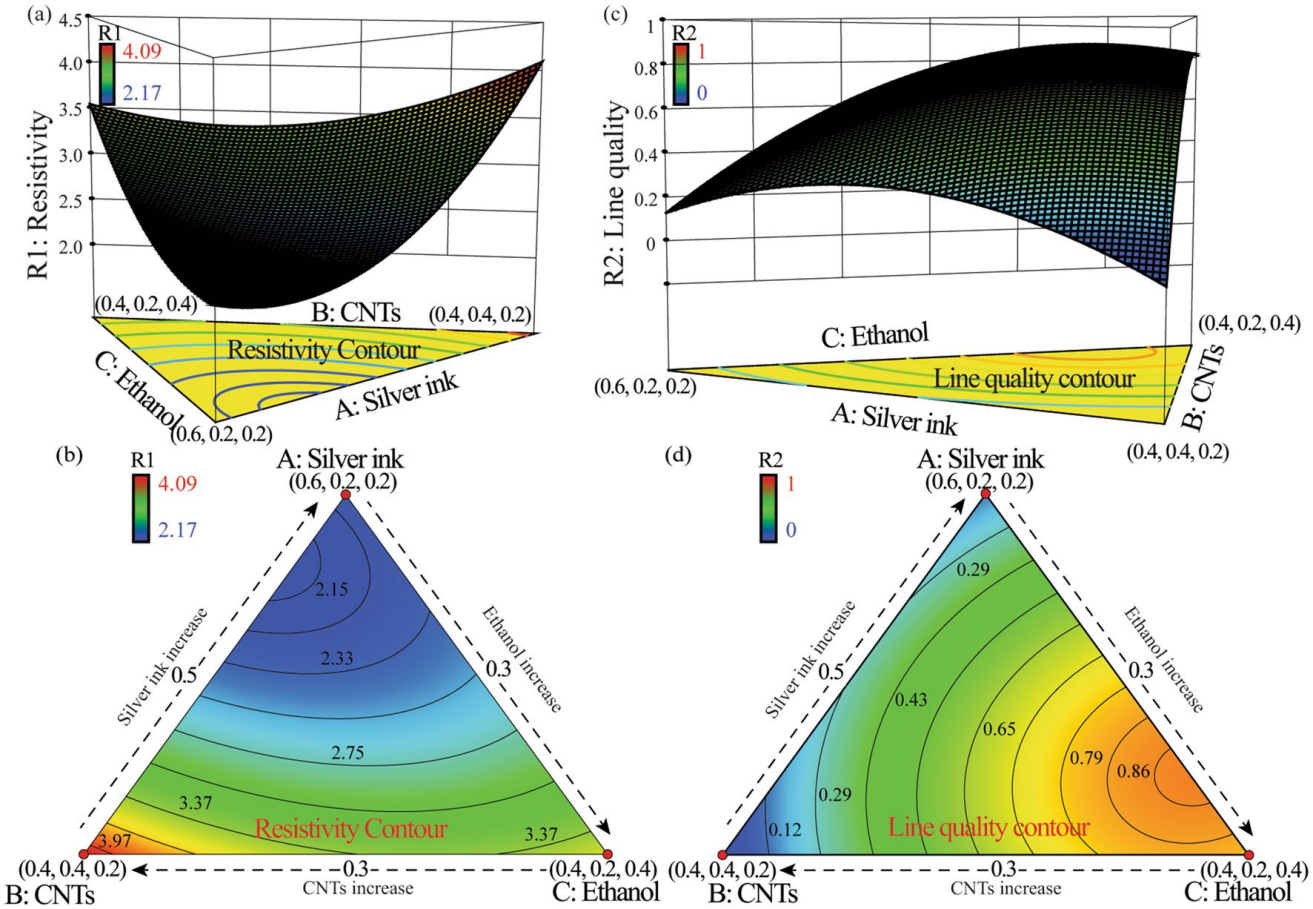
The obtained responses and the corresponding contours for (**a**, **b**) printed line resistivity and (**c**, **d**) printed line quality, respectively.

In this research, CNTs and ethanol are incorporated into original functional inks to enhance printed line conductivity and printed line quality, respectively. However, as the volume fraction of each composite cannot be changed independently, it is crucial to determine an optimal operating material window in the mixture design space to get a balance between low printed line resistivity and high printed line quality simultaneously. Specifically, Fig. 5a, b demonstrates that the replacement level of CNTs less than 0.25 will has a positive influence on the printed line conductivity. This is because the incorporated CNTs bundles can serve as conductive bridges to connect the small cracks (see Supplementary Fig. S7a) in the deposited line (see Supplementary Fig. S7b, c), which will be beneficial to the improvement of printed line conductivity^40^. However, further increasing the volume fraction of CNTs, the insufficient silver nanoparticle material will be divided into discontinued blocks (see Supplementary Fig. S7d), thereby reducing the inter-particle connectivity and gradually increasing the printed line resistivity.

Likewise, to enhance the printing quality, it is necessary to keep the volume fraction of ethanol around 0.35 while minimizing the replacement of CNTs (Fig. 5c, d). Specifically, the lines will be printed with high overspray or high edge roughness (see Supplementary Fig. S8a, b) due to the high concentrations of the atomized nanoparticles^12^. Despite incorporating ethanol into the functional ink helps to enhance the ink printability (see Supplementary Fig. S8c), further increasing the volume fraction of ethanol may result in insufficient component of silver nanoparticles and excessive diffusion of aerosol on the substrate (see Supplementary Fig. S8d). Therefore, it is necessary to make a compromise when trying to capture the high printed line quality and low printed line resistivity simultaneously^41^.

Based on a desirability function approach, the optimal material composition is identified to optimize the conflicting responses in a mixture design space. In this research, the weights of printed line resistivity and printed line quality in the overall desirability function can be selected according to users’ preference, which were set to be 0.6 and 0.4 in this study, respectively. Moreover, the lower/higher limit of the printed line resistivity and printed line quality were set as $\left[2.17, 4.09\right]$ µΩ⋅cm and $\left[0, 1\right]$, respectively. In this case, the obtained overall desirability is 0.902, which is promising according to previous research^42^, and the identified optimal fractions of the three composites (silver ink, CNTs ink and ethanol) are 0.502, 0.219 and 0.279, respectively.

**Figure 6:**
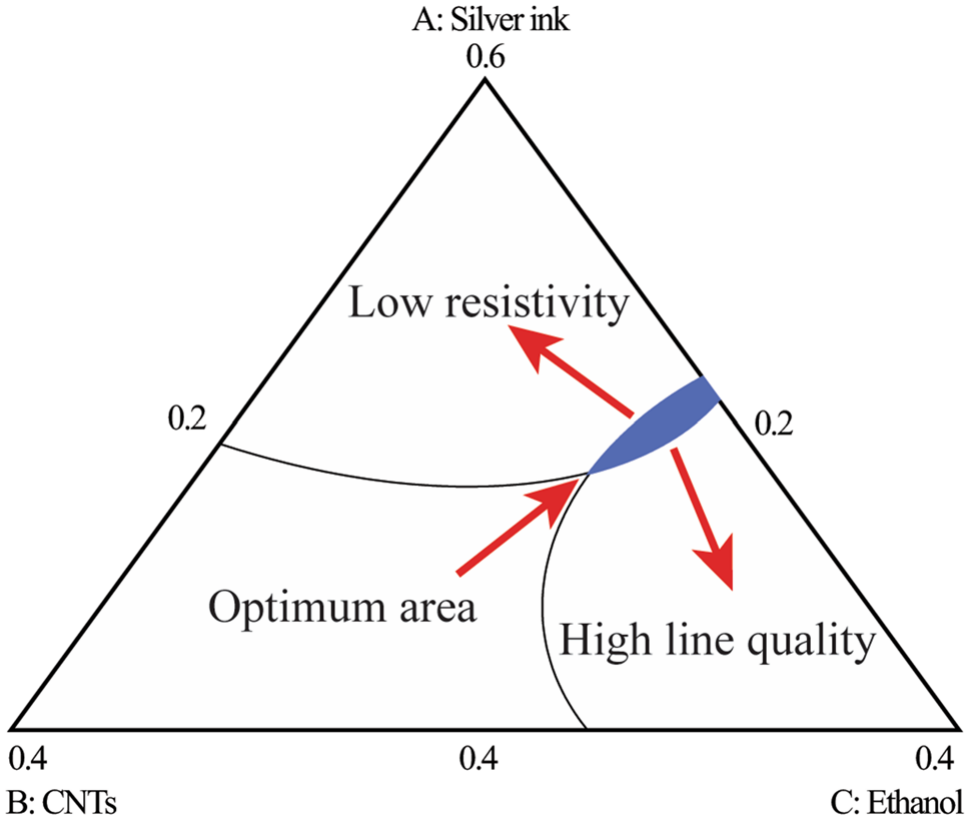
The identified optimal operating material window in a mixture design space.

To further determine an optimal operating window of material composition, the identified optimal fractions of the three composites is set as a reference point in a mixture design space. Then, the critical contour of each target response is acquired by setting an optimization threshold of this reference point in an interval of a%, i.e., reference ± a%. Thereafter, the overlapping region of the critical contours is identified as an optimal operating material window to capture the low printed line resistivity and high printing quality simultaneously. In this research, we set a% to be 4%, and the identified optimal operating material window is shown in Fig. 6.

**Figure 7:**
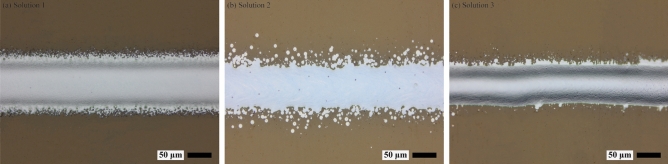
Deposited line characteristics obtained according to the selected ink compositions. (**a, b**) Outside the optimum area, (**c**) inside the optimal operating material window.

As the comparison between the best samples and worst samples obtained may not fully validate the effectiveness of the determined optimal operating window, the ink compositions were randomly selected from and outside the optimal operating window for further verification. Table 4 describes the selected ink compositions (randomly chosen from and outside the optimal operating window) and the corresponding printed line resistivity/quality, Fig. 7a–c illustrates the deposited line characteristics obtained according to the selected ink compositions (Solution 1–Solution 3) from Table 4, respectively. The experiments indicate that the determined optimal operating material window is conducive to get a balance between low printed line resistivity and high printed line quality, demonstrating the validity of the adopted optimization method in AJP.

**Table 4:** Details of the selected ink compositions (volume fraction).

Solutions	Silver ink	CNTs ink	Ethanol	Resistivity (µΩ⋅cm)	Line quality
Solution 1	0.42	0.25	0.33	3.26	0.81
Solution 2	0.51	0.26	0.23	2.29	0.46
Solution 3	0.49	0.22	0.29	2.51	0.72

### Optimizing functional ink composition in the 3D mixture design space via a NSGA-III algorithm and statistical uncertainty

**Figure 8:**
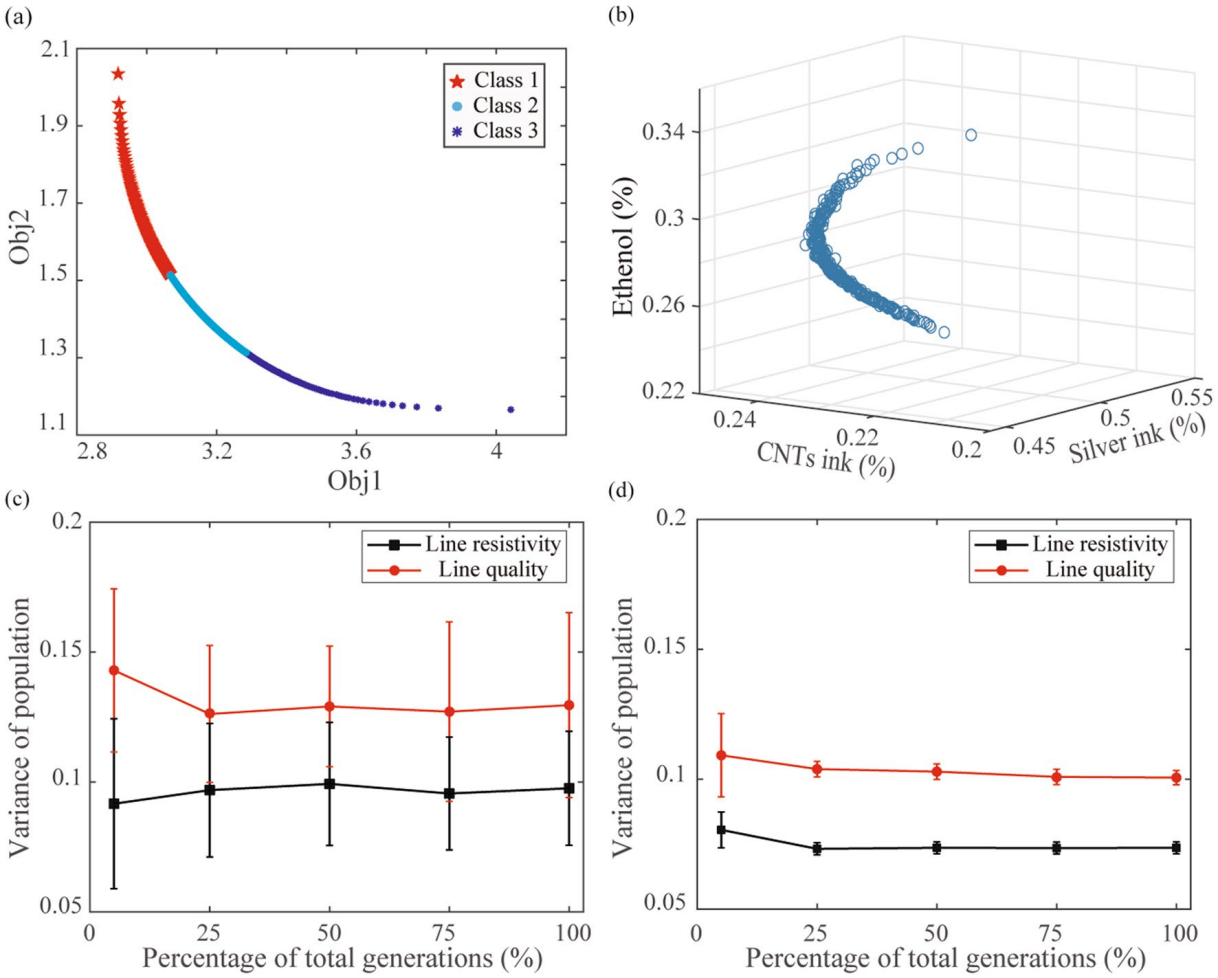
(**a**) The Pareto front obtained by considering the uncertainty of statistical prediction, (**b**) the obtained Pareto-optimal solution set corresponding to (**a**). (**c**) The variance of population obtained by optimizing only ReSMs, (**d**) the variance of population obtained by integrating ReSMs with prediction uncertainty.

Figure 8a, b shows the obtained Pareto front and the corresponding Pareto-optimal solution set, respectively. The obtained Pareto-optimal solution set will be considered as candidate solutions in the mixture design space. However, it will be computationally or experimentally expensive to verify the obtained entire Pareto-optimal points during the optimization process. Therefore, affinity propagation (AP) clustering approach is adopted to identify clustering centroids of the Pareto-optimal solution set as representative solutions for further verification^43^. Moreover, since the statistical uncertainty is considered in the objective functions, NSGA-III tends to identify a set of chromosomes that can simultaneously minimize statistical uncertainty and target outputs during evolution. Therefore, Fig. 8c, d shows that the overall variance of the obtained populations is much lower than the corresponding optimization without considering the prediction uncertainty, indicating the improved robustness of the identified Pareto-optimal solution set during evolution.

**Figure 9:**
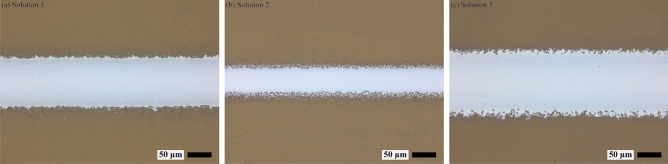
(**a**–**c**) Deposited line morphology achieved using the representative functional ink compositions in Table 5 respectively.

The selected three representative functional ink components and the corresponding printed line characteristics are shown in Table 5 and Fig. 9, respectively. Table 5 indicates that a group of Pareto-optimal points rather than a single solution are identified to balance the conflicting relationship between low printed line resistivity and high printed line quality. Therefore, different selections can be made according to the users’ preference, which helps to optimize the conflicting targets more objectively. Different from previous studies where similar resistivity was obtained in pure silver lines^44^, the mechanical flexibility and the morphological characteristics of printed lines can be improved simultaneously in this research due to the incorporation of CNT and ethanol.

**Table 5:** Representative functional ink compositions based on NSGA-III.

Solutions	Silver ink	CNTs ink	Ethanol	Resistivity (µΩ⋅cm)	Line quality
Solution 1	0.45	0.23	0.32	2.91	0.91
Solution 2	0.52	0.23	0.25	2.32	0.65
Solution 3	0.48	0.24	0.28	2.63	0.79

On the other hand, as CNTs have the advantages of superior mechanical flexibility^45^, bending test experiments were conducted to confirm the bridging conduction effect of CNTs. As shown in Fig. S9a–c, based on the mechanical stage and motion control system of an aerosol jet 3d printer, the thermistors printed with optimized nanocomposites and original functional ink were subjected to repeated bending test under the bending radius of 6 mm (1.04% strain), respectively. Compared with the original functional ink (see Fig. S9d), the bending test results show that the printed line conductivity is enhanced due to the bridging conduction effect of CNTs (see Fig. S9e). However, it should be noted that the overall relative resistance may vary slightly because of the small cracks formed in the nanocomposites after the prolonged bending test (see Fig. S9f).

In this research, the functional ink composition (nanoparticle silver ink, CNTs ink and ethanol) was optimized based on the proposed hybrid multi-objective optimization approach. The determined optimal functional ink can achieve a printed resistivity of 2.3 µΩ⋅cm, which was 32% lower than the pure silver lines, and was 1.45 times of the bulk silver resistivity. Despite comparable printed line resistivity can be obtained by post-processing^9–11^, it is important to enhance the intrinsic connectivity within the nanoparticle structure via mixture design. Moreover, as the proposed approach optimize the printed line resistivity and printed line quality simultaneously, it is more efficient than traditional multi-layer printing. This is because the traditional method may reduce the controllability of printed line morphology due to the complex interaction between successive layers.

## Conclusions

In this research, the cause-effect relationship between different ink components (nanoparticle silver ink, CNTs ink and ethanol) and the printing results were systemically investigated based on the proposed hybrid multi-objective optimization approach, and the functional ink composition was optimized to achieve low electrical resistivity and high printed line quality, which can be helpful to improve the electrical performance of the microelectronic devices produced by AJP. As AJP technology is compatible with a wider range of functional inks and constituent nanoparticle sizes, there has been increasing interest in using AJP technology for fabricating versatile micro-electronic devices. In this case, the data-driven characteristics facilitate the optimization of multiple conflicting properties of ink compositions in a data-driven manner, which will be beneficial to the development of multi-functional microelectronic components and the wide application of AJP technology in the field of printed electronics.

However, unlike printing straight lines, the printed curve lines may deform due to stress concentration. Therefore, it may be necessary to adjust the main process parameters in the process of printing curve lines to alleviate the stress concentration in further study. In addition, the factors that affect the overlapping quality of adjacent lines, such as plate temperature, print speed and overlapping ratio will also be systematically studied, which helps to improve the quality of the printed patterns in the case of very fine features. Moreover, due to the highly nonlinear interaction between the ink compositions and the printing process parameters, adjusting the process parameters according to specific ink properties can further improve the printing results. Therefore, it is important and challenging to simultaneously optimize the multiple conflicting properties within multi-space in future research work.

## Supplementary Information

Supplementary Information.

## Data Availability

The datasets generated and analyzed during the current study are available from the corresponding author on reasonable request.
