# Supplementary material for: A hybrid multi-objective optimization of functional ink composition for aerosol jet 3D printing via mixture design and response surface methodology
Source: Sci Rep. 2023 Feb 13;13:2513. doi: 10.1038/s41598-023-29841-0 (PMC9925446; doi:10.1038/s41598-023-29841-0)
Supplement: Supplementary file 1 — Supplementary Information. [file 41598_2023_29841_MOESM1_ESM.pdf]

Supplementary Information

A hybrid multi-objective optimization of functional ink composition for aerosol jet 3D printing via mixture design and response surface methodology

Haining Zhang[1,2], Zhixin Liu[3], Shuai Yin[2], Haifeng Xu[1,*]

[1]School of Information Engineering, Suzhou University, Suzhou 234000, China

[2]School of Mechanical and Aerospace, Nanyang Technological University, Singapore, 639798

[3]China Aerospace Times Feihong Technology Co., Ltd., 100854 Beijing, China

*__Corresponding author__

Address: School of Information Engineering, Suzhou University, Suzhou 234000, China

E-mail: hf.xu.su@gmail.com

S1. Evaluation of printing repeatability and the influence of coffee-ring effect

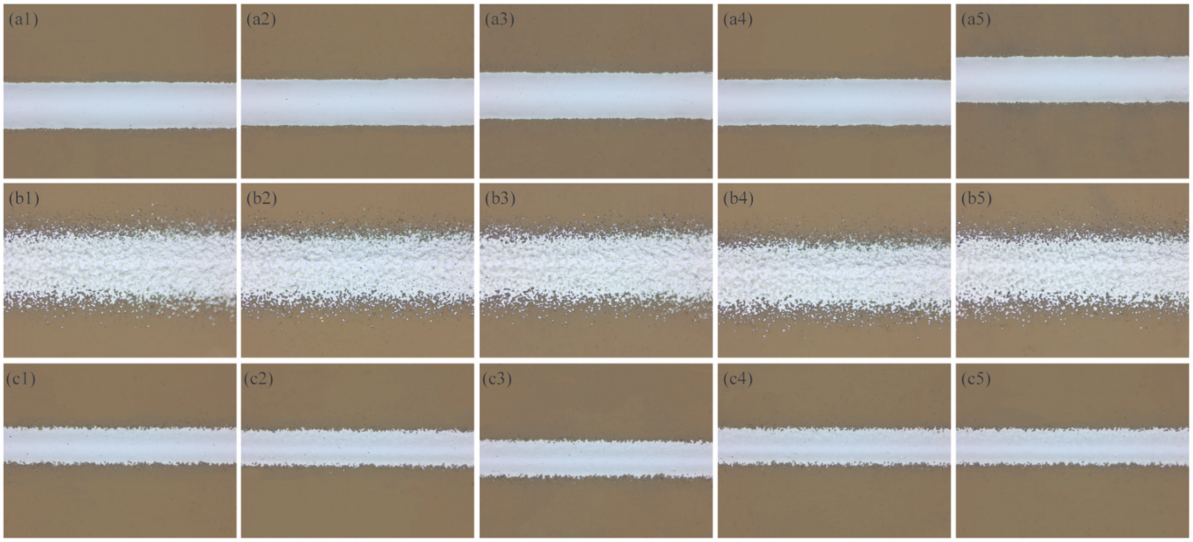

Fig. S1. Evaluation the repeatability of the printed line morphology under different ink compositions. (a1-a5) Less overspray and better edge definition lines deposited for 5 times, (b1-b5) high overspray and high edge roughness lines deposited for 5 times, and (c1-c5) high edge roughness lines deposited for 5 times.

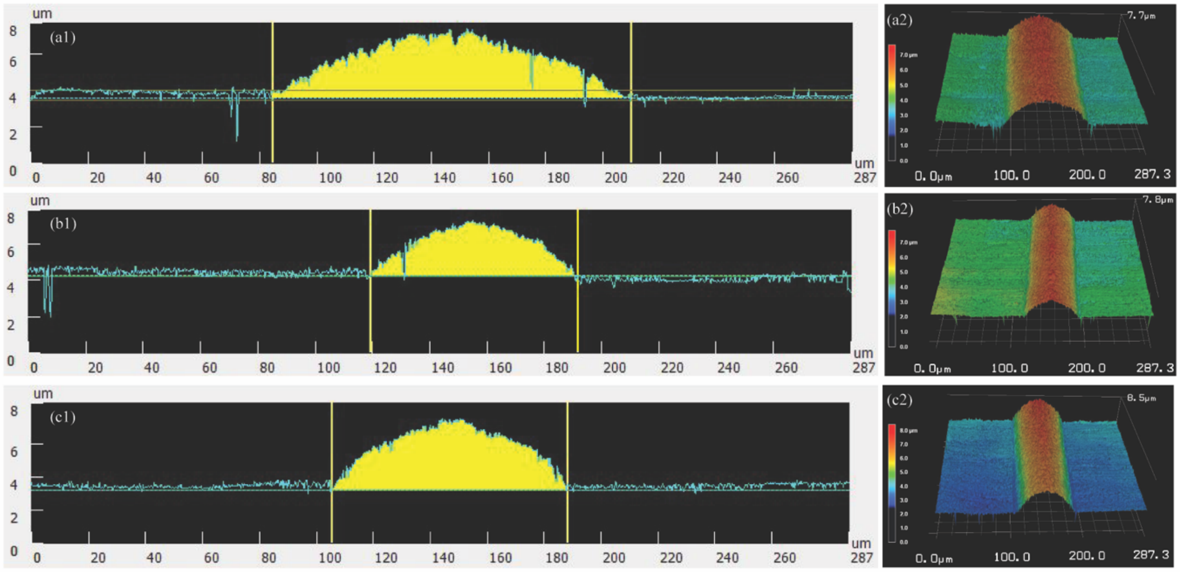

Fig. S2. Measured cross-sectional profiles of printed lines 4 hours after deposition at room temperature.

S2. Morphological characterization of printed line samples

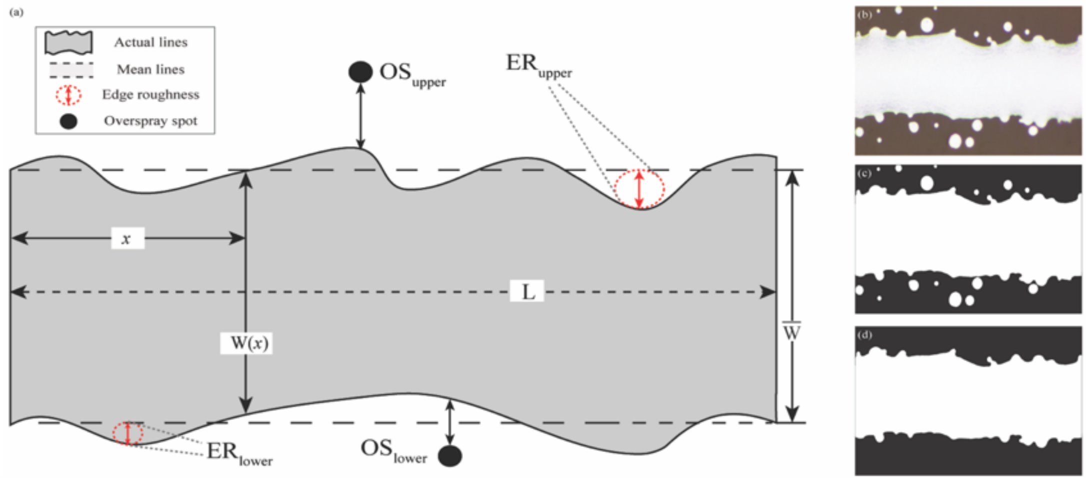

Fig. S3. Morphological characterization and extraction of deposited line samples. (a) Illustration of a line sample deposited with high overspray and edge roughness, (b) original image of a printed line sample, (c) obtained printed line morphology with overspray, (d) obtained printed line morphology with denoising.

No.	Microscopic images	Line quality	No.	Microscopic images	Line quality
1	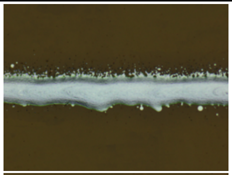	0.29	5	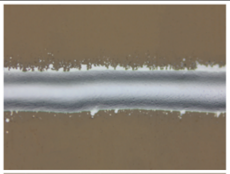	0.71
2	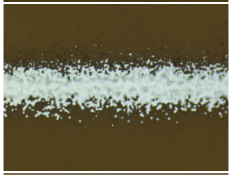	0.36	6	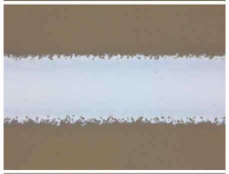	0.76
3	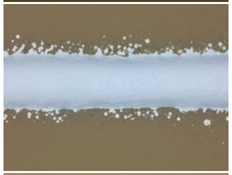	0.53	7	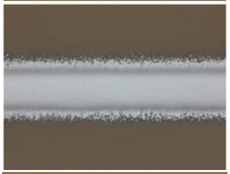	0.81
4	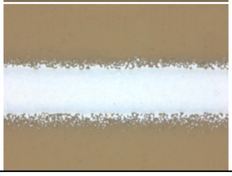	0.63	8	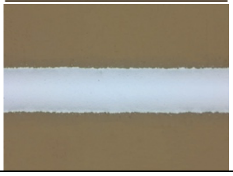	1

Table S1. The representative microscopic images of printed lines and the corresponding quantified printed line quality.

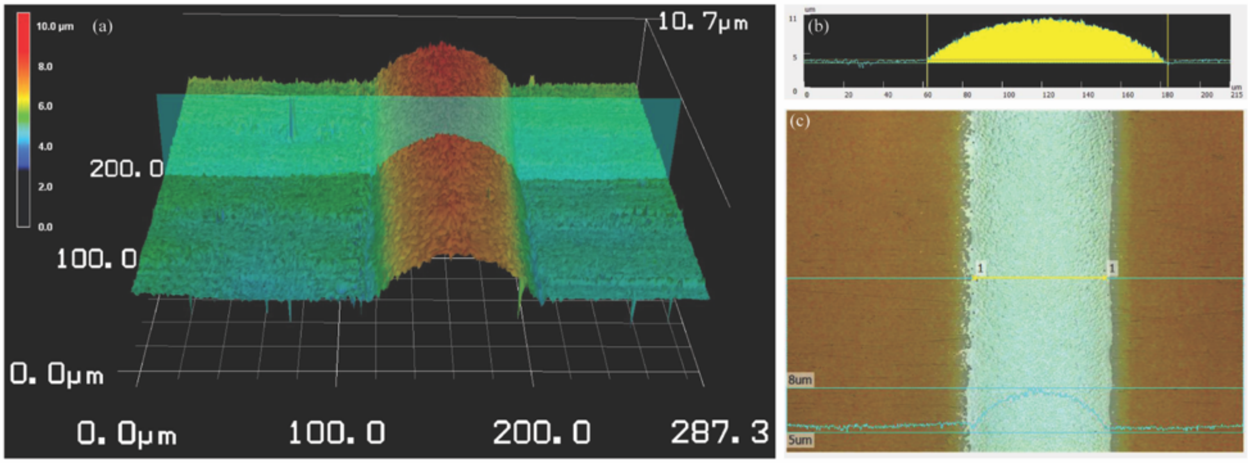

Fig. S4. 3D profile extraction of AJP. (a) Extracted 3D profile of a deposited line sample, (b) cross-section extraction of a deposited line sample, (c) the deposited line sample corresponding to (a).

S3. The designed mixture components

No.	Silver ink	CNTs	Ethanol (%)	Viscosity (cP)
1	0.400	0.400	0.200	2.71
2	0.400	0.200	0.400	2.25
3	0.400	0.300	0.300	2.58
4	0.433	0.333	0.233	3.14
5	0.433	0.233	0.333	2.95
6	0.433	0.283	0.283	3.05
7	0.483	0.233	0.283	3.43
8	0.483	0.283	0.233	3.58
9	0.500	0.200	0.300	3.62
10	0.467	0.267	0.267	3.34
11	0.500	0.300	0.200	4.54
12	0.600	0.200	0.200	4.58
13	0.533	0.233	0.233	3.89

Table S2. The designed mixture components and the actual volume fractions.

S4. Multi-objective optimization based on NSGA-III

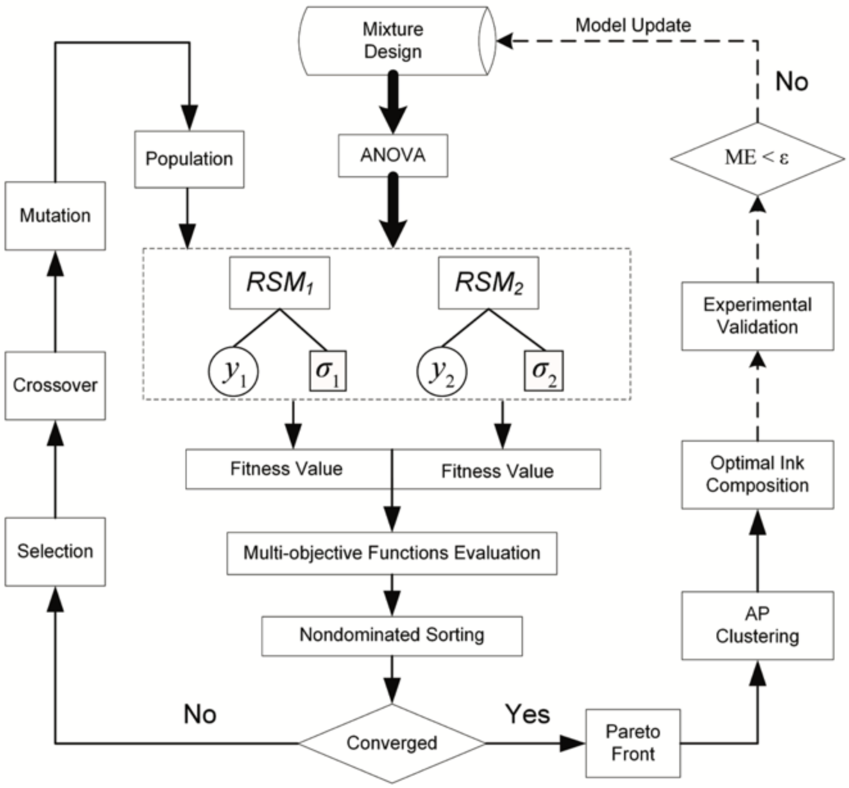

Fig. S5. The proposed flow chart for optimizing functional ink composition in 3D mixture design space.

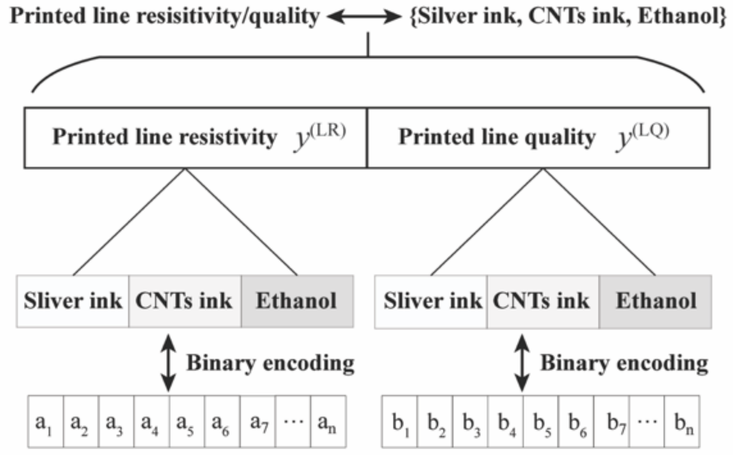

Fig. S6. The proposed chromosome encoding form for multi-objective optimization.

Parameters	Settings
Number of RSMs	2
Threshold of modeling error	10%
Number of input parameters	3
Objective functions	2
(1) $\text{Obj}_1 = y^{(LR)} + \lambda_1 \sigma^{(LR)}$	$\lambda_1 = 1.6$
(2) $\text{Obj}_2 = \frac{1}{y^{(LQ)}} + \lambda_2 \sigma^{(LQ)}$	$\lambda_2 = 2.9$
Constraints of adjustable parameters	4
(1) Silver ink (%)	[0.4, 0.6]
(2) CNTs ink (%)	[0.2, 0.4]
(3) Ethanol (%)	[0.2, 0.4]
(4) Silver ink + CNTs ink + Ethanol = 1	Volume fraction
Number of clusters	3
Mutation probability	0.01
Population size	300
Maximal generations	600
Crossover probability	0.9

Table S3. System settings for multi-objective optimization.

Based on the adopted system settings, the obtained Pareto-optimal solution set will be considered as candidate solutions in the mixture design space, and the derived RSMs will be calibrated with additional experiments if the modeling error (ME) exceeds the threshold ε.

$$\text{ME} = \frac{1}{n}\sum_{i=1}^{n}\frac{|\hat{y}_i - y_i|}{y_i} \times 100\% \quad \text{(S1)}$$

where $\hat{y}_i$ is the predictive output response, y_i is the measured printed line resistivity/quality.

S5. Analysis of printed line samples

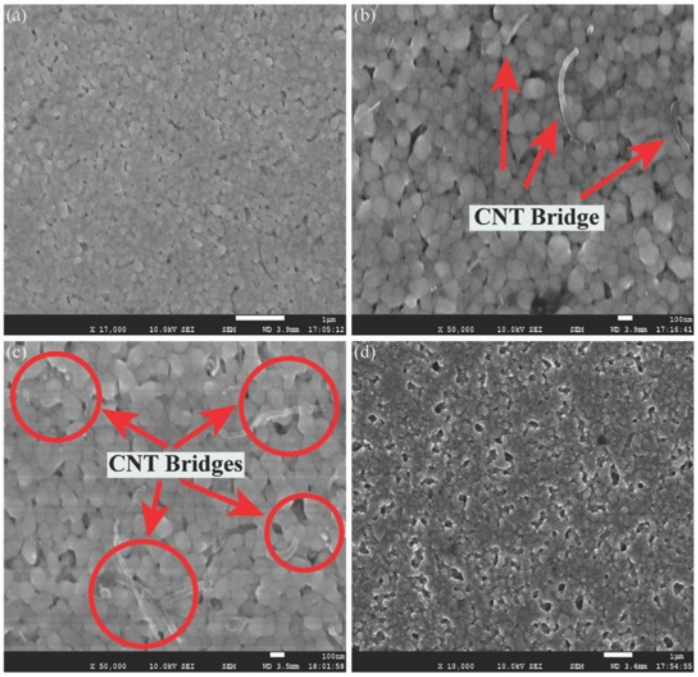

Fig. S7. Scanning electron microscope images of a (a) printed silver line, (b) printed sliver/CNTs line, (c) printed silver line with increased CNTs, (d) printed line with insufficient silver material.

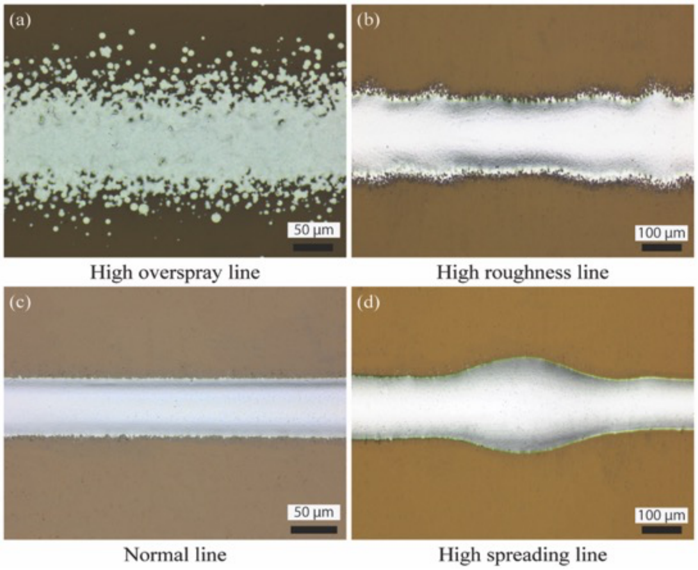

Fig. S8. Comparison of different morphological features. (a) High overspray line, (b) high edge roughness line, (c) normal line, (d) high spreading line.

S6. Bending test

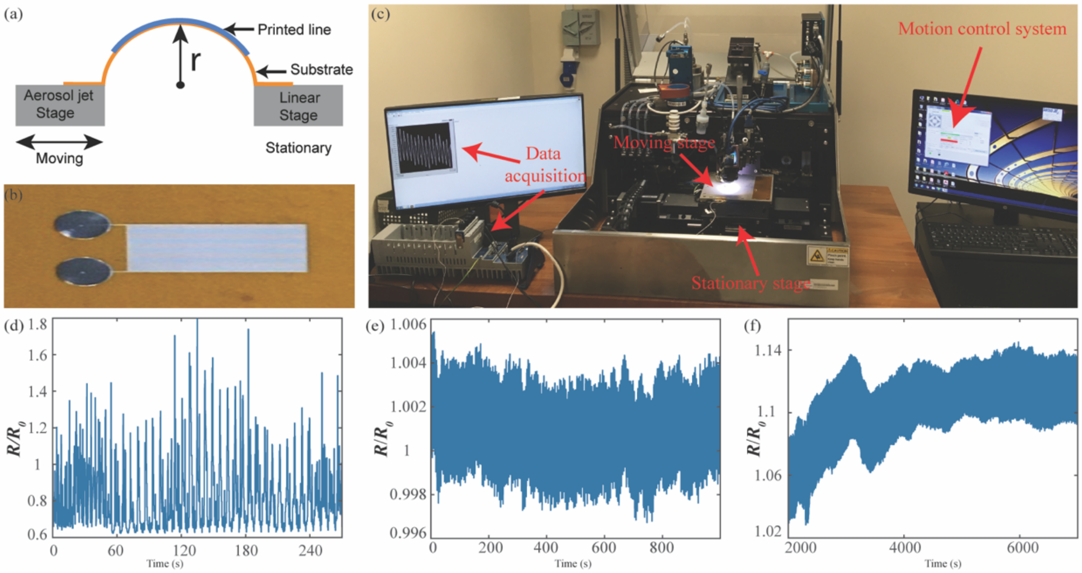

Fig. S9. Bending test experiments. (a) Schematic depicting the experimental method for bending test, (b) printed thermistor for bending test, (c) experimental setup for bending test. Bending test results of printed (d) silver thermistor, (e) sliver/CNTs thermistor. (f) The overall relative resistance variation of sliver/CNTs thermistor after the prolonged bending test.
